# Sepsis-Induced Coagulopathy and Hypoalbuminemia: Endothelial Damage as Common Pathway and Clinical Implications on Mortality and Transfusion Risk

**DOI:** 10.3390/jcm14134483

**Published:** 2025-06-24

**Authors:** Gianni Turcato, Arian Zaboli, Fabrizio Lucente, Lucia Filippi, Michael Maggi, Gloria Brigiari, Paolo Ferretto, Alessandro Cipriano, Lorenzo Ghiadoni, Christian J. Wiedermann

**Affiliations:** 1 Intermediate Care Unit, Department of Internal Medicine, Hospital Alto Vicentino (AULSS7), 36014 Santorso, Italy; fabrizio.lucente@aulss7.veneto.it (F.L.); lucia.filippi@aulss7.veneto.it (L.F.); michael.maggi@aulss7.veneto.it (M.M.); paolo.ferretto@aulss7.veneto.it (P.F.); 2 Department of Health Sciences, UniCamillus-Saint Camillus International University of Health Sciences, 00131 Rome, Italy; 3 Innovation, Research and Teaching Service (SABES-ASDAA), Teaching Hospital of the Paracelsus Medical Private University (PMU), 39100 Bolzano, Italy; zaboliarian@gmail.com; 4 Unit of Biostatistics, Epidemiology and Public Health, Department of Cardiac, Thoracic, Vascular Sciences and Public Health, University of Padova, 35122 Padova, Italy; gloria.brigiari@ubep.unipd.it; 5 Emergency Department, Nuovo Santa Chiara Hospital, Azienda Ospedaliero-Universitaria Pisana, 56126 Pisa, Italy; alessandrocipriano@gmail.com; 6 Department of Clinical and Experimental Medicine, University of Pisa, 56126 Pisa, Italy; lorenzo.ghiadoni@unipi.it; 7 Institute of General Medicine and Publich Health, Claudiana, 39100 Bolzano, Italy; christian.wiedermann@am-mg.claudiana.bz.it

**Keywords:** sepsis, sepsis-induced coagulopathy, serum albumin, endothelial dysfunction, capillary leak syndrome

## Abstract

**Background:** Sepsis-induced coagulopathy (SIC) and hypoalbuminemia represent distinct yet interrelated manifestations of endothelial dysfunction in sepsis. While both have been individually associated with increased mortality, their combined prognostic value remains unexplored. This study aimed to assess the relationship between the SIC score and serum albumin levels and to evaluate their integrated role in predicting mortality and bleeding risks in septic patients. **Methods:** We conducted a prospective observational study enrolling adult patients with community-acquired sepsis admitted to an Intermediate Medical Care Unit between January 2023 and June 2024. The primary outcome was 30-day all-cause mortality. The secondary outcome was the occurrence of ISTH-defined major bleeding. Multivariable logistic regression and Net Reclassification Improvement (NRI) analyses were performed to evaluate the predictive value of albumin when added to the SIC score. **Results:** A total of 413 patients were enrolled; 18.4% had a positive SIC score. The serum albumin and SIC score were inversely correlated (r = −0.189, *p* < 0.001). Both variables were independently associated with 30-day mortality and major bleeding. The addition of albumin significantly improved the predictive performance of the SIC score (NRI = 0.276 for mortality; NRI = 0.268 for bleeding; both *p* = 0.003). The cluster analysis identified distinct phenotypes based on albumin and SIC profiles, with differing clinical trajectories and transfusion needs. **Conclusions:** The combined assessment of the SIC score and serum albumin enhances early risk stratification in sepsis. This dual-parameter approach may support more accurate prognostication and individualized management in septic patients.

## 1. Introduction

Sepsis-induced coagulopathy (SIC) is an early marker of the dysregulated host response that defines sepsis [1,2]. The SIC score, introduced by the International Society on Thrombosis and Haemostasis (ISTH), combines platelet counts, the international normalized ratio (INR), and the Sequential Organ Failure Assessment (SOFA) score, with values ≥4 indicating a clinically relevant coagulopathy [1,2,3]. Although initially subclinical, SIC may progress to overt-disseminated intravascular coagulation and has been recognized as an independent predictor of short-term mortality in patients with sepsis [1,2,3].

The development of SIC is closely linked to early endothelial dysfunction induced by inflammatory mediators. Endothelial injury disrupts the vascular balance between coagulation and anticoagulation, promoting tissue factor expression, impairing physiological anticoagulant pathways, inhibiting fibrinolysis, and increasing vascular permeability [2,4,5,6]. This culminates in a systemic prothrombotic state, characterized by microthrombi formation, the consumption of platelets and coagulation factors, and impaired tissue perfusion [2,5,6,7]. 

In parallel, capillary leak syndrome emerges as another consequence of endothelial damage. The structural disruption of the glycocalyx and intercellular junctions compromises vascular integrity, allowing plasma proteins, particularly albumin, to extravasate into the interstitial space [8,9,10]. The resulting hypoalbuminemia is not only a marker of vascular leakage but also contributes to interstitial edema, reduced oncotic pressure, and impaired oxygen delivery, all of which are associated with worse outcomes [8,11]. 

Although SIC and hypoalbuminemia represent different clinical phenomena, both derive from a shared pathophysiological substrate, endothelial injury, and are independently associated with an increased mortality in sepsis. However, their interrelationship has never been systematically investigated, and it remains unclear whether they develop concurrently, influence one another, or reflect distinct pathways within the spectrum of endothelial dysfunction.

## 2. Study Objectives

(a) To analyze the relationship between the risk of coagulopathy, assessed through the SIC score, and capillary permeability, as reflected by serum albumin levels;
(b) To explore the joint distribution of these two variables within the study population using a cluster analysis;
(c) To assess the potential net benefit of incorporating serum albumin levels into the SIC score for predicting 30-day mortality, major bleeding risks, and transfusion requirements.

## 3. Methods

### 3.1. Study Design and Setting

This is a prospective observational study conducted from 1 January 2023 to 30 June 2024 in the Intermediate Medical Care Unit (IMCU) of the General Medicine Department at Alto Vicentino Hospital in Santorso, Italy.

The IMCU is located within the internal medicine ward and includes 12 beds, a dedicated team of physicians and nurses, and advanced equipment for the monitoring and management of critically ill patients.

The unit is designed to provide high-intensity care, including invasive monitoring of vital parameters and non-invasive ventilation. In addition, advanced procedures are routinely performed for the management of central venous catheters and continuous infusion therapies, with the possibility of administering inotropic and vasopressor agents for hemodynamic support.

Unlike standard medical wards, the IMCU combines enhanced monitoring capacity with an overall higher treatment intensity. This approach enables more controlled patient stabilization and a more efficient allocation of healthcare resources within the department.

### 3.2. Patients

During the study period, all patients diagnosed with sepsis and admitted to the IMCU were considered eligible for enrollment. Sepsis was defined according to current international guidelines as a suspected or confirmed infection associated with a SOFA score of ≥2 [12]. Exclusion criteria were as follows: (I) age under 18 years; (II) lack of informed consent; (III) suspected or confirmed pregnancy; (IV) admission from departments other than the Emergency Department; (V) sepsis secondary to surgery or trauma within the previous three months; (VI) ED stay exceeding six hours; (VII) terminal illness with an expected survival of less than three months; and (VIII) patients who would have required immediate organ support (e.g., orotracheal intubation), but for whom such interventions were not performed due to chronic or palliative care decisions precluding ICU admission.

Patients with partially or entirely incomplete clinical documentation relevant to the study were also excluded to ensure the inclusion of only those with complete medical records and all necessary study data.

Informed consent was obtained from all eligible patients. When a patient was unable to provide consent, it was obtained from a next of kin or legal guardian.

### 3.3. Study Protocol

At admission, for all enrolled patients, both a physician and a nurse collected medical history and clinical information including age, sex, body mass index (BMI), and comorbidities (hypertension, ischemic heart disease, peripheral vascular disease, stroke, chronic heart failure, diabetes, chronic kidney disease, cancer, and antithrombotic therapy). These data were used to calculate the Charlson Comorbidity Index (CCI). BMI was also analyzed as an indirect marker of nutritional status, considering its potential relevance to baseline albumin levels and their association with sepsis-related outcomes. This allowed us to explore the possible role of malnutrition in modulating both hypoalbuminemia and coagulation disturbances. Vital signs were also recorded at the time of admission, both as individual parameters and aggregated into the National Early Warning Score (NEWS).

Simultaneously, a venous blood sample was taken. The samples were analyzed to determine the following:

(1) Inflammatory markers: C-reactive protein (CRP), white blood cells (WBCs) and procalcitonin (PCT);
(2) Coagulation parameters: platelet count, PT-INR, aPTT, Fibrinogen, and D-Dimer;
(3) Complete blood count: hemoglobin (Hb) and hematocrit (Hct);
(4) Creatinine;
(5) Bilirubin;
(6) Albumin.

Based on these data, the Acute Physiology and Chronic Health Evaluation (APACHE) II score and SOFA score were calculated.

Subsequently, sepsis-induced coagulopathy (SIC) was assessed using PT-INR, platelet count, and SOFA score [13]. The maximum SIC score is 6, with a value ≥4 considered indicative of SIC [13].

For all enrolled patients, the SIC score was calculated using data obtained at the time of admission to the IMCU. This time point was chosen to ensure consistency and reflect the early clinical presentation of sepsis prior to therapeutic interventions.

All physicians involved in conducting this study participated in several training sessions focused on this study’s objectives and patient management in accordance with the current sepsis guidelines. This approach aimed to standardize patient care as much as possible and minimize the degree of subjectivity.

### 3.4. Outcomes

The primary outcome of the study was 30-day all-cause mortality, confirmed through consultation with the local civil registry.

The secondary outcome was the occurrence of an ISTH-defined major bleeding event, defined according to ISTH criteria as a transfusion need of at least two blood units, corresponding to the definition of major bleeding [14].

### 3.5. Statistical Analysis

Continuous variables were expressed as means with standard deviations (SDs) or medians with interquartile ranges (IQRs), depending on their distribution as assessed by the Shapiro–Wilk test. Categorical variables were presented as absolute numbers and percentages. Group comparisons (e.g., SIC-positive vs. SIC-negative; survivors vs. non-survivors; patients with vs. without an ISTH-defined major bleeding event) were performed using Student’s *t*-test or Mann–Whitney U test for continuous variables and the Chi-squared test or Fisher’s exact test for categorical variables, as appropriate.

A descriptive heatmap was generated to visualize the percentage distribution of patients across SIC score values and serum albumin levels, using clinically relevant cut-offs. This graphical representation aimed to explore the overlap between coagulopathy and capillary leak profiles and to identify the most prevalent clinical combinations at presentation.

Associations between continuous variables, such as serum albumin and SIC score or the number of transfused units, were analyzed using Pearson’s correlation coefficient (r).

Multivariable logistic regression models were used to identify independent predictors of 30-day mortality and major bleeding. Given the limited number of SIC events (n = 76), we applied a variable selection strategy to avoid overfitting, ensuring an event-per-variable ratio of at least 8:1. Only the most relevant predictors were retained in the final model based on clinical relevance and statistical significance. Results were reported as adjusted odds ratios (ORs) with 95% confidence intervals (CIs).

To assess the improvement in prognostic accuracy from adding serum albumin to the SIC score, the Net Reclassification Improvement (NRI) was calculated for both 30-day mortality and major bleeding outcomes. The NRI was assessed separately for events and non-events and tested for significance using the Z-statistic.

Additionally, an unsupervised cluster analysis was performed to identify patient subgroups based on SIC score, serum albumin, and clinical severity (SOFA and APACHE II scores). Differences across clusters were evaluated using one-way ANOVA or Kruskal–Wallis tests for continuous variables and Chi-squared tests for categorical variables. Kaplan–Meier survival analysis was performed as a visual tool to compare 30-day survival across clusters. However, given the fixed 30-day follow-up and consistency with other models, logistic regression was used for inferential statistics.

All analyses were conducted using STATA 16.1 (StataCorp LLC, College Station, TX, USA). A *p*-value < 0.05 was considered statistically significant.

### 3.6. Ethical Consideration

This study was approved by the local ethics committee (Clinical Trial Ethics Committee ULSS 8, Berica-Vicenza, Italy; approval number: 19814; approval date: 1 June 2022) and was conducted in accordance with the ethical principles for medical research involving human subjects as defined by the Declaration of Helsinki. Informed consent was obtained from all eligible patients. In cases where the patient was unable to provide consent, it was sought from the next of kin or legal guardian.

## 4. Results

A total of 413 patients were enrolled during the study period. The mean SIC score recorded upon admission to the IMCU was 3.4 (±1.2), with 18.4% of patients (76/413) presenting a positive SIC score. Patient characteristics are detailed in Supplementary Table S1.

Compared to patients with a negative SIC score, those with a positive SIC score exhibited a significantly lower BMI, a higher incidence of active malignancies, more pronounced alterations in coagulation parameters (including PT, INR, and aPTT), lower platelet counts, and more severe anemia (Supplementary Table S1). Additionally, the SIC-positive group showed a lower systolic blood pressure, a higher oxygen saturation, reduced white blood cell counts, and significantly higher SOFA scores (Supplementary Table S1). Serum albumin was not found to be an independent risk factor for SIC positivity, as confirmed by both the multivariate logistic regression and multivariate linear analysis for the SIC score prediction (Supplementary Table S1).

The mean serum albumin level in the overall cohort was 2.6 g/dL (±0.5). At the IMCU admission, the mean albumin level was 2.6 g/dL (±0.5) in patients with a negative SIC score and 2.5 g/dL (±0.6) in those with a positive SIC score (*p* = 0.056; Supplementary Table S1). Serum albumin showed a statistically significant negative correlation with the SIC score, with a Pearson correlation coefficient of −0.189 (*p* < 0.001).

The percentage distribution of patients according to SIC score values and serum albumin levels at admission is illustrated in Supplementary Figure S1. The 2.5–3.0 g/dL albumin range was the most represented, peaking at a SIC score of three (11.4%), followed by the 2.0–2.5 g/dL range (9.9%). As the SIC score increases, the proportion of patients decreases within each albumin category. Combinations involving albumin levels < 1.5 g/dL or >3.5 g/dL were marginally represented (<1%).

Overall, patients were predominantly distributed in an intermediate zone, with albumin between 2.0 and 3.0 g/dL and SIC scores between 2 and 4, suggesting a frequent overlap between moderate coagulopathy and hypoalbuminemia.

The overall 30-day mortality rate in the study cohort was 16.9% (70/413). The characteristics of survivors and non-survivors at 30 days are summarized in Table 1.

The mean serum albumin level across the cohort was 2.6 g/dL (±0.5), and 87.2% of patients (360/413) had albumin levels below the normal reference range (<3.5 g/dL), indicating a high prevalence of hypoalbuminemia at presentation.

Patients who died were significantly older, had a higher Charlson Comorbidity Index, and more frequently presented with active cancer. They also showed a lower systolic blood pressure and hemoglobin levels, along with increased D-dimer, creatinine levels, and higher SOFA and APACHE II scores. Oxygen saturation, heart rate, and coagulation parameters were also worse in the non-survivor group (Table 1).

Patients who survived at 30 days had significantly higher mean serum albumin levels compared to non-survivors (2.65 ± 0.51 vs. 2.27 ± 0.46 g/dL, *p* < 0.001; Table 2).

Similarly, the mean SIC score was lower among survivors than non-survivors (3.3 ± 1.1 vs. 3.8 ± 1.2, *p* < 0.001; Table 1). In the multivariate logistic regression analysis adjusted for variables associated with 30-day mortality, both the SIC score and serum albumin were identified as independent predictors of adverse outcomes. Specifically, higher SIC scores were associated with an increased risk of death (OR: 1.405; 95% CI 1.096–1.800; *p* = 0.007), whereas higher albumin levels showed a protective effect (OR 0.401; 95% CI 0.215–0.749; *p* = 0.004).

The potential prognostic improvement obtained by incorporating serum albumin into the SIC score is reported in Table 2.

The addition of serum albumin values to the baseline predictive model based on the SIC score resulted in a significant improvement in the ability to predict 30-day mortality, with an NRI of 0.276 (Z = 2.91, *p* = 0.003; Table 2). This value indicates that albumin contributed to a correct net reclassification of 27.6% of patients, enhancing the predictive accuracy of the SIC score (Table 2). Among the patients who died (n = 70), 30 were correctly reclassified to a higher risk category, while 7 were incorrectly reclassified to a lower risk category (Table 2). Overall, in 32.8% (23/70) of non-survivors, albumin improved the predictive power of the SIC score. Among survivors (n = 343), 96 patients were reclassified to a higher risk group and 78 to a lower risk group, resulting in a net worsening in predictions of 5.2% (18/343).

The cluster analysis identified three distinct patient clusters. The characteristics of these clusters are presented in Table 2.

The three clusters identified through the cluster analysis revealed patient groups with significantly different serum albumin levels and SIC scores, suggesting the presence of clinically distinct phenotypes (Table 3).

Cluster 1 was characterized by reduced mean albumin levels (2.3 g/dL) and high SIC scores (4.6), indicative of a severe inflammatory and coagulopathic condition (Table 4).

In contrast, Cluster 2 showed higher albumin concentrations (3.1 g/dL) and lower SIC scores (2.9), representing a more stable clinical profile (Table 3). Finally, Cluster 3 presented with albumin levels similar to Cluster 1 (2.3 g/dL) but with lower SIC scores (2.5), suggesting a dissociated pattern between capillary leakage and coagulation activation (Table 3).

With regard to the clinical severity and outcomes, the three clusters exhibited distinct trajectories. Cluster 1 was associated with elevated SOFA and APACHE II scores (5.4 and 14.1, respectively) and a 30-day mortality rate of 25.9% (37/143). Cluster 2 showed lower SOFA and APACHE II scores and a 30-day mortality rate of 7.9% (12/152) (Table 3). Cluster 3 displayed intermediate values for both the SOFA (3.7) and APACHE II (13.1) and a mortality rate of 17.8% (21/118).

**Figure 1:**
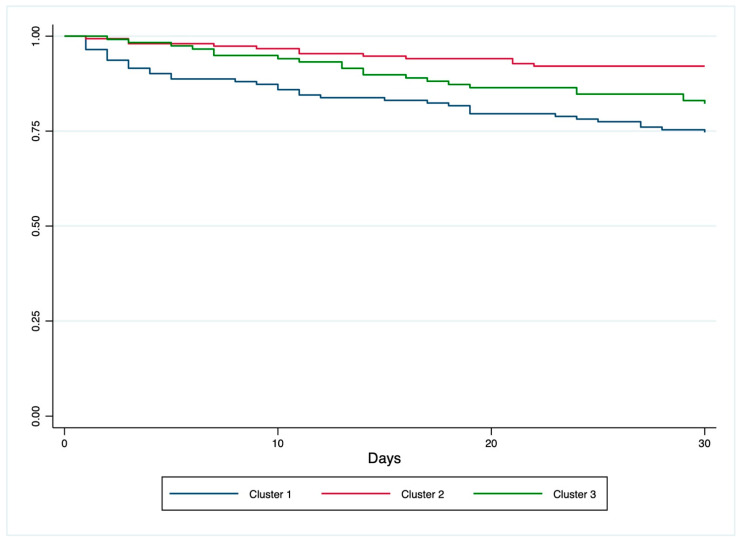
Kaplan–Meier survival curves for 30-day mortality stratified by patient clusters. The differences in survival between the three clusters were statistically significant (Log-Rank test, *p* < 0.001).

The Kaplan–Meier analysis presented in Figure 1 demonstrated statistically significant differences in the survival across the three clusters (Log-Rank *p* < 0.001).

Additionally, 24.5% of the cohort (101/413) received at least one transfusion within 30 days of admission, while 14.8% (61/413) experienced an ISTH-defined major bleeding event. The characteristics of these patients are reported in Supplementary Table S2.

The most frequent bleeding sites were associated with consumption or microangiopathic processes (44.3%), followed by gastrointestinal bleeding (18.1%) and hematologic disease-related bleeding (18.1%). The full distribution is detailed in Supplementary Table S3.

Patients who experienced an ISTH-defined major bleeding event had significantly lower BMIs, hemoglobin, and hematocrit levels (Supplementary Table S2). These patients also showed a significant increase in PTL, PT-INR, and aPTT values. The presence of an active malignancy was more frequent in those with bleeding (41% vs. 15.3%, *p* < 0.001). Both SOFA and APACHE II scores were significantly higher in the bleeding group.

Patients with an ISTH-defined major bleeding event had significantly lower serum albumin levels compared to those without bleeding (2.32 ± 0.45 vs. 2.63 ± 0.52 g/dL, *p* < 0.001) and a higher mean SIC score (3.9 ± 1.1 vs. 3.3 ± 1.3, *p* < 0.001; Supplementary Table S2). In the multivariate analysis adjusted for clinical variables associated with bleeding risks in the univariate analysis, the SIC score was independently associated with an increased risk of major transfusion (adjusted OR 1.535; 95% CI 1.153–2.045; *p* = 0.003), whereas serum albumin showed a trend toward a protective effect (adjusted OR 0.524; 95% CI 0.270–1.014; *p* = 0.055).

The number of transfused units showed a statistically significant inverse correlation with the admission serum albumin (Pearson’s r = −0.234, *p* < 0.001) and a positive correlation with the SIC score (Pearson’s r = +0.224, *p* < 0.001).

The potential improvement in the bleeding risk prediction from the addition of serum albumin to the SIC score is presented in Table 4.

The inclusion of serum albumin in the SIC score-based prediction model yielded an NRI of 0.268 (Z = 2.92, *p* = 0.003), indicating a statistically significant overall improvement of 26.8% in the model’s ability to reclassify the risk of major bleeding. Among patients who experienced major bleeding, 19 were correctly reclassified to a higher risk category, whereas 8 were incorrectly reclassified to a lower risk, resulting in a net benefit in 18.1% of patients (11/61; Table 4). Among patients without major bleeding, the addition of albumin correctly reclassified 90 individuals to a lower risk category, while 59 were incorrectly shifted to a higher risk. Overall, 8.8% of patients (31/352) were correctly reclassified due to the inclusion of serum albumin (Table 4).

When stratified by previously identified clusters, Cluster 1 exhibited a major bleeding rate of 23.8% (34/143), compared to 7.9% (12/152) in Cluster 2 and 12.7% (15/118) in Cluster 3 (*p* = 0.001). Regarding transfusion requirements, 39.9% (57/143) of patients in Cluster 1 required at least one transfusion, compared to 11.8% (18/152) in Cluster 2 and 22% (26/118) in Cluster 3 (*p* < 0.001).

## 5. Discussion

This study, conducted on a large prospective cohort of patients with community-acquired sepsis, is the first to systematically explore the relationship between the SIC score and serum albumin levels, considered readily accessible clinical surrogates of sepsis-induced coagulopathy and capillary leak, respectively. These two markers were found to be correlated, suggesting that the alteration of one is frequently associated with changes in the other, although no direct causal relationship was demonstrated. Their combined use significantly enhanced the predictive ability for both 30-day mortality and the occurrence of ISTH-defined major bleeding events. The cluster analysis further identified distinct clinical phenotypes, reinforcing the notion that the degree of the capillary permeability alteration and hemostatic dysfunction may differentially contribute to the clinical trajectory of septic patients.

During sepsis, the intense cytokine activation rapidly targets the vascular endothelium, which acts as a central regulator of both metabolic and immune responses [15,16]. An endothelial injury impairs the ability to maintain coagulation homeostasis and vascular barrier integrity [17,18], leading to systemic coagulopathy characterized by microthrombi formation, the consumption of coagulation factors, and erythrocyte destruction [18]. Concurrently, the increased capillary permeability, driven by the glycocalyx degradation, tight junction disruption, and inflammatory vasodilation, facilitates the leakage of fluids and plasma proteins, particularly albumin, contributing to the capillary leak phenomenon and impaired organ perfusion [19]. Serum albumin and the SIC score, indirect indicators of capillary leaks and sepsis-induced coagulopathy, respectively, have been extensively but independently studied as short-term mortality predictors in sepsis [20,21].

To the best of our knowledge, this is the first study to investigate the relationship between the SIC score and serum albumin levels, based on the hypothesis of a shared pathophysiological origin and the independent association of both markers with mortality in septic patients. This study offers several novel insights with potential implications for clinical practice.

First, the SIC score and serum albumin levels exhibit a correlated pattern in patients with sepsis; when one parameter is altered, the other tends to be altered as well. However, despite this correlation, they do not appear to exert a direct causal influence on each other. This finding supports the notion that sepsis-induced coagulopathy and capillary leaks are epiphenomena of a common underlying process, namely, endothelial dysfunction [10]. During sepsis, a systemic inflammatory activation leads to early endothelial injury, involving glycocalyx degradation, endothelial activation, and tissue factor expression, ultimately resulting in the disruption of the hemostatic balance [16,22]. This cascade promotes uncontrolled thrombin generation, the inhibition of physiological anticoagulant pathways (including protein C, thrombomodulin, and antithrombin), and the suppression of fibrinolysis [3,4,5]. The net effect is the formation of microthrombi and the progressive development of consumption coagulopathy and microangiopathic hemolysis [3,4,5]. At the same time, glycocalyx degradation and progressive endothelial cell dysfunction increase vascular permeability, facilitating the extravasation of fluids and plasma proteins such as albumin [16,18,20,23]. In addition to albumin, the glycocalyx degradation during sepsis contributes to the non-selective leakage of other mid-sized plasma proteins, including endogenous anticoagulants such as antithrombin and protein C. This loss, secondary to the endothelial junction disruption, may further exacerbate coagulopathy and microvascular thrombosis [23,24]. The result is an increase in interstitial edema and likely alterations in the extracellular matrix itself, with serious consequences for tissue and organ perfusion [16,18,20,23]. In our study, although no direct causal relationship was identified between the two mechanisms, markedly low serum albumin levels appeared to be associated with an increased risk of bleeding and coagulopathy. In the study by Rinaldi et al., serum albumin levels ≤ 2.5 g/dL were significantly associated with a higher risk of DIC, with an OR of 2.363 (95% CI: 1.201–4.649) [25]. In the study by Ebina et al., an antithrombin supplementation in septic patients significantly reduced early mortality only among those with serum albumin levels < 2.5 g/dL, while no significant benefit was observed in patients with albumin levels ≥ 2.5 g/dL [26].

Second, this study confirms what is already well established, that the SIC and serum albumin are independent predictors of mortality. However, it also suggests for the first time that their combined use can enhance the prediction of disease severity by effectively integrating two critical pathophysiological processes that coexist during sepsis. While the SIC score remains a practical bedside tool, it does not capture the loss of antithrombin, a key marker of a poor prognosis in sepsis. Prior studies have shown that incorporating antithrombin levels into DIC scoring systems improves the risk stratification and prediction of ICU mortality [27]. This suggests that, although pragmatic, the SIC score may provide an incomplete picture of the coagulation profile in septic patients. Given that SIC combined with hypoalbuminemia may identify a particularly high-risk subgroup, it could be hypothesized that an early antithrombin supplementation, thanks to its anti-inflammatory and endothelial-stabilizing properties, might offer clinical benefits in selected patients. This is in line with current emerging evidence on antithrombin’s pleiotropic effects [27,28]. Although additional markers such as antithrombin III and protein C/S may provide deeper insight into the pathophysiology of sepsis-induced coagulopathy, they are not routinely available in acute care settings due to cost and logistical constraints. Our study focused instead on standard parameters that are readily accessible, to ensure clinical applicability in real-world environments. To our knowledge, no similar data have been previously published in the literature. Li et al. demonstrated that patients with persistent SIC beyond day four had a significantly higher 28-day mortality rate (HR 3.736, 95% CI: 2.025–6.891), with the SIC score performing comparably to the SOFA score in terms of predictive accuracy [29]. Similarly, Wang et al. reported that patients with a SIC score ≥ 4 had an in-hospital mortality rate of 47.3%, compared to 29.5% in other patients, with an odds ratio of 2.15 (95% CI: 1.29–3.59) for the risk of death [30]. In the study by Schmoch et al., it was suggested that the presence of coagulopathy may be associated with both impaired organ perfusion (as reflected by elevated SOFA scores) and tissue hypoperfusion (evidenced by hyperlactatemia), anticipating a potential link between these underlying pathophysiological conditions [31]. Serum albumin has been widely recognized as a risk factor for mortality in septic patients. Arnau-Barres et al. showed that serum albumin levels <2.6 g/dL were strongly associated with an increased 30-day mortality (OR 3.26; 95% CI: 1.12–9.41; *p* = 0.029) [32], while Yin et al. reported a significantly lower survival among patients with albumin levels < 29.2 g/L compared to those with levels ≥ 29.2 g/L [33]. Albumin has also demonstrated strong potential in improving the predictive performance of the SOFA score in two previous studies, a finding that mirrors our results regarding the significant prognostic reclassification obtained by incorporating albumin into the SIC score [34,35].

Third, low serum albumin levels and elevated SIC scores were significantly associated with an increased risk of requiring blood transfusions, including ISTH-defined major bleeding events. The integration of albumin into the SIC score improved its predictive performance for transfusion risk. While the SIC score has been extensively validated as a mortality predictor, few studies have investigated its correlation with actual blood product utilization in clinical practice. Tullio et al. demonstrated that a positive SIC score upon an ED admission independently predicted major bleeding events (OR 4.83), thrombotic complications (OR 9.48), and the need for transfusions (OR 5.28) [36]. In a prior study, Wang et al. also suggested a role for albumin in predicting transfusion needs: patients with hypoalbuminemia (<29.2 g/L) received a significantly higher mean number of transfused units compared to those with albumin ≥ 29.2 g/L (4.04 ± 6.76 vs. 2.14 ± 4.36, *p* < 0.001), and the overall transfusion rate was higher in the hypoalbuminemic group (58.2% vs. 40.1%, *p* < 0.001) [30]. The assessment of bleeding risks and transfusion requirements in septic patients could benefit substantially from the incorporation of serum albumin levels into the standard SIC-based risk stratification. In our study, the addition of albumin enhanced the SIC score’s discriminatory power, with an NRI of 26.8% (*p* = 0.003), indicating a significant improvement in bleeding risk classification. These findings fill a gap in the current literature and suggest that a combined evaluation of coagulopathy and capillary leak may provide a more accurate and individualized approach to hemorrhagic risk assessments in patients with sepsis. Additionally, although D-dimer is a widely used biomarker of fibrinolytic activation and coagulopathy in sepsis, its levels did not consistently differ between subgroups in our analysis. This may be explained by the study design: only patients admitted directly from the Emergency Department were enrolled, resulting in a cohort predominantly composed of individuals in the early stages of sepsis, before extensive endothelial or fibrinolytic activation had occurred. As such, D-dimer may not yet fully capture the extent of fibrinolysis or microvascular thrombosis at the time of measurement in this clinical context.

## 6. Limitations

First, it was conducted at a single center, which may limit the generalizability of the findings.

Second, in the multivariate analyses, variables already included in the calculation of the SIC score were excluded to avoid collinearity and redundancy. Similarly, hemoglobin and hematocrit were not included in models predicting transfusion requirements.

Third, no specific analysis of DIC was performed. However, since DIC at admission is often clinically indistinguishable from SIC, the SIC score was considered a suitable surrogate for sepsis-induced coagulopathy. Additionally, the use of transfusion as an objective outcome more accurately reflects real-world clinical practice.

Fourth, no data on thrombotic events within 30 days were available, limiting the interpretation of the pro-thrombotic aspects of coagulopathy. A potential limitation of this study lies in the interpretation of the SIC score, which may be affected by clinical factors not directly related to sepsis, such as chronic liver disease, baseline thrombocytopenia, or ongoing anticoagulant therapy. While these variables could theoretically influence score components and introduce a degree of misclassification, it is important to note that the SIC score was specifically developed and validated in heterogeneous real-world populations where such confounders are commonly present. Therefore, its use remains clinically relevant and reflective of routine bedside assessments. Moreover, certain chronic conditions can alter baseline coagulation parameters, potentially leading to elevated SIC scores in the absence of acute sepsis-related coagulopathy. These factors were partially captured in our dataset and described in Supplementary Table S1; however, their presence may still represent a source of diagnostic uncertainty and should be considered when interpreting SIC values in complex patients.

Fifth, it is possible that some critically ill patients or those who died early did not receive transfusions due to clinical decisions regarding therapeutic limitations or the rapidity of their clinical deterioration. This reflects the observational, real-life nature of this study, though it is believed that such occurrences did not significantly affect the overall results.

We were unable to account for the fluid balance in the present cohort and thus could not quantify the potential contribution of hemodilution to anemia or transfusion requirements. Additionally, as the SIC score was assessed only at admission, the dynamic changes in the coagulation status during hospitalization were not captured. While this approach reflects an early risk stratification, it does not account for temporal fluctuations in SIC. Moreover, data on isolated pathogens and administered therapies (e.g., antibiotics or anticoagulants) were not systematically collected in this cohort and were therefore not analyzed. This limits the possibility of correlating specific etiologies or treatment strategies with clinical outcomes. Finally, total serum protein levels were not available for this cohort and could not be analyzed. Although our study focused on serum albumin as a specific marker of endothelial dysfunction and capillary leakage, the lack of total protein data limits the ability to assess whether hypoalbuminemia reflected a selective albumin loss or a more generalized reduction in circulating proteins.

## 7. Conclusions

In this prospective cohort study of patients with sepsis, serum albumin and the SIC score emerged as independent and complementary predictors of the clinical severity, 30-day mortality, and bleeding risk. Their correlation reflects the coexistence of endothelial dysfunction, capillary leak, and sepsis-induced coagulopathy, two key pathophysiological processes in sepsis. The integration of serum albumin into the SIC score significantly improved its prognostic accuracy for both mortality and transfusion-related outcomes. These findings suggest that combining readily available laboratory markers reflecting distinct, yet interconnected, aspects of the sepsis pathophysiology can enhance risk stratification and support a more personalized clinical approach.

Further multicenter studies are warranted to validate these findings and to explore the potential utility of this combined approach in guiding early therapeutic decisions and improving outcomes in septic patients.

## Figures and Tables

**Table 1:** Baseline characteristics of the study population stratified by 30-day mortality. SIC: sepsis-induced coagulopathy. BMI: body mass index. CHF: congestive heart failure. CKD: chronic kidney disease. RR: respiratory rate. SpO_2_: peripheral oxygen saturation. HR: heart rate. SBP: systolic blood pressure. NEWS: National Early Warning Score. CRP: C-reactive protein. WBC: white blood cell count. PLT: platelet count. PT-INR: prothrombin time–international normalized ratio. aPTT: activated partial thromboplastin time. Hb: hemoglobin. Hct: hematocrit. SOFA: Sequential Organ Failure Assessment. APACHE II: Acute Physiology and Chronic Health Evaluation II.

Variables	Alive at 30 Days	Dead at 30 Days	*p*-Value
Patients, n (%)	343 (83.1)	70 (16.9)	
Age, years, mean (SD)	70.7 (12.9)	75.8 (9.6)	0.001
Sex, n (%)			0.498
Male	130 (37.9)	23 (32.9)	
Female	213 (62.1)	47 (67.1)	
BMI, kg/m^2^, median (IQR)	25.3 (22.8–29.4)	24.7 (21.9–26.8)	0.105
Comorbidities, n (%)			
Hypertension	214 (62.4)	50 (71.4)	0.173
Ischemic heart disease	43 (12.5)	11 (15.7)	0.442
Peripheral vascular disease	54 (15.7)	12 (17.1)	0.724
Stroke or TIA	31 (9)	3 (4.3)	0.237
Chronic heart failure	45 (13.1)	17 (24.3)	0.026
Diabetes	82 (24.3)	19 (28.4)	0.536
Chronic kidney failure	60 (17.5)	14 (20)	0.610
Active cancer	52 (15.2)	27 (38.6)	<0.001
Charlson Comorbidity Index, point, mean (SD)	4.6 (2.5)	6.3 (2.5)	<0.001
Antithrombotic therapy, n (%)			
Antiplatelet	60 (17.5)	15 (21.4)	0.494
Anticoagulant	100 (29.2)	20 (28.6)	1.000
Type of anticoagulant, n (%)			0.874
Direct oral anticoagulants	89 (89)	17 (85)	
Vitamin K antagonist	7 (7)	2 (10)	
Low-molecular-weight heparin	4 (4)	1 (5)	
Site of infection, n (%)			0.005
Genitourinary	82 (23.9)	8 (10.8)	
Respiratory	145 (42.4)	28 (40)	
Intra-abdominal	36 (10.6)	9 (12.3)	
Musculoskeletal/skin	24 (7)	2 (3.1)	
Other	8 (2.4)	1 (1.5)	
Unknown/viral	48 (13.6)	22 (32.3)	
Vital signs			
RR, breaths/min, median (IQR)	20 (18–25)	20 (18–25)	0.284
SpO_2_, %, median (IQR)	95 (93–98)	94 (90–97)	0.043
HR, bpm, median (IQR)	90 (80–108)	96 (80–108)	0.219
Systolic BP, mmHg, mean (SD)	115.6 (25.7)	102.8 (22.8)	<0.001
NEWS, point, mean (SD)	5.1 (3.3)	7.3 (3.4)	<0.001
Inflammatory markers, median (IQR)			
CRP, mg/dL	14.8 (8.1–22.3)	16.1 (7.4–22.4)	0.881
WBC, ×10^9^/L	12.4 (7.5–17.9)	11.2 (6.7–16.6)	0.303
PCT, ×10^9^/L	4.5 (0.9–21)	3.9 (0.9–16.2)	0.416
Coagulation parameters, median (IQR)			
Platelet count, ×10^9^/L	173 (124–251)	131 (77–270)	0.053
PT-INR	1.25 (1.14–1.39)	1.33 (1.21–1.48)	0.004
aPTT	1.22 (1.09–1.41)	1.26 (1.08–1.44)	0.375
Fibrinogen, mg/dL	542 (427–745)	503 (362–660)	0.083
D-Dimer, ng/mL	2056 (1035–4100)	3546 (1465–6217)	0.008
Complete blood count			
Hb, g/dL, mean (SD)	12.1 (2.6)	11.3 (2.5)	0.017
Hct, %, median (IQR)	0.37 (0.32–0.42)	0.34 (0.29–0.39)	0.005
Creatinine, mg/dL, median (IQR)	1.43 (0.99–2.17)	1.78 (1.19–2.52)	0.037
Bilirubin, mg/dL, median (IQR)	0.9 (0.5–1.6)	1.1 (0.7–1.6)	0.195
Albumin, g/dL, mean (SD)	2.65 (0.51)	2.27 (0.46)	<0.001
SIC score, point, mean (SD)	3.3 (1.1)	3.8 (1.2)	<0.001
Severity scores, mean (SD)			
SOFA score	3.9 (1.6)	5.4 (2.5)	<0.001
APACHE II score	12.1 (4.6)	15.7 (5.1)	<0.001

**Table 2:** The NRI comparing the predictive performance of the SIC score alone versus the combined model including serum albumin for 30-day mortality. The table shows the distribution of patients reclassified into different risk categories after adding serum albumin to the SIC score, separately for non-survivors and survivors. Bold values indicate patients whose risk was incorrectly reclassified (worse classification), while underlined values indicate a correct reclassification (improved classification).

Net Reclassification Index					
Deceased	SIC + Albumin				
SIC	<10%	10–20%	20–30%	>30%	Total
<10%	3	2	4	1	10
10–20%	**2**	20	7	11	40
20–30%	**1**	**3**	3	5	12
>30%	**0**	**0**	**1**	7	8
Total	6	25	15	24	70
Alive	SIC + Albumin				
SIC	<10%	10–20%	20–30%	>30%	Total
<10%	67	**30**	**6**	**2**	105
10–20%	56	84	**32**	**17**	189
20–30%	3	13	7	**9**	32
>30%	1	3	2	11	17
Total	127	130	47	39	343

**Table 3:** Clinical characteristics and outcomes of patient clusters identified through the unsupervised analysis. Clusters were derived based on the SIC score and serum albumin levels at admission. Cluster 1 was characterized by low albumin (mean 2.3 g/dL), a high SIC score (mean 4.6), and the highest rate of SIC positivity (48.3%). Cluster 2 displayed the most favorable profile, with preserved albumin levels (mean 3.1 g/dL), low SIC scores (mean 2.9), and minimal SIC positivity (4.6%). Cluster 3 showed hypoalbuminemia similar to Cluster 1 (mean 2.3 g/dL), but lower SIC scores (mean 2.5).

Variables	Cluster 1	Cluster 2	Cluster 3	*p*-Value
Patients, n (%)	143 (34.6)	152 (36.8)	118 (28.6)	
Age, years, mean (SD)	72.9 (11.3)	70.2 (14.7)	71.6 (11.1)	0.180
Albumin, mg/dL, mean (SD)	2.3 (0.4)	3.1 (0.3)	2.3 (0.3)	<0.001
SIC score, point, mean (SD)	4.6 (0.8)	2.9 (0.8)	2.5 (0.5)	<0.001
Positive SIC, n (%)	69 (48.3)	7 (4.6)	0 (0.0)	<0.001
SOFA score, point, mean (SD)	5.4 (2.1)	3.5 (1.3)	3.7 (1.5)	<0.001
APACHE score, point, mean (SD)	14.1 (0.4)	10.9 (4.5)	13.1 (4.7)	<0.001
30-day mortality, n (%)	37 (25.9)	12 (7.9)	21 (17.8)	<0.001

**Table 4:** The NRI comparing the predictive performance of the SIC score alone versus the combined model including serum albumin for ISTH-defined major bleeding events. The table shows the distribution of patients reclassified into different risk categories after adding serum albumin to the SIC score, separately for patients with and without an ISTH-defined major bleeding event. Bold values indicate patients whose risk was incorrectly reclassified (worse classification), while underlined values indicate correct reclassifications (improved classification).

Net Reclassification Improvement					
ISTH-Defined Major Bleeding Event	SIC + Albumin				
SIC	<10%	10–20%	20–30%	>30%	Total
<10%	5	4	0	0	9
10–20%	**4**	18	7	2	31
20–30%	**1**	**2**	3	6	12
>30%	**0**	**0**	**1**	8	9
Total	10	24	11	16	61
No ISTH-Defined Major Bleeding Event	SIC + Albumin				
SIC	<10%	10–20%	20–30%	>30%	Total
<10%	83	**22**	**1**	**0**	106
10–20%	68	101	**24**	**5**	198
20–30%	2	15	8	**7**	32
>30%	0	4	1	11	16
Total	154	142	34	23	352

## Data Availability

Data available on request due to privacy/ethical restrictions.

## Supplementary Materials

The following supporting information can be downloaded at https://www.mdpi.com/article/10.3390/jcm14134483/s1, Figure S1: A heatmap showing the percentage distribution of patients according to the SIC score and serum albumin levels categorized by clinical cut-offs. Each cell represents the proportion (%) of patients within a specific SIC score (*x*-axis) and albumin range (*y*-axis). The figure highlights the frequent overlap between moderate hypoalbuminemia and intermediate SIC scores, suggesting a clustering of patients in the mid-range of both variables. The color intensity is proportional to the relative frequency of patients in each cell; Table S1: Baseline characteristics of the study population stratified by SIC status. SIC: sepsis-induced coagulopathy; Table S2: Baseline characteristics of the study population stratified by the presence of ISTH-defined major bleeding events; Table S3: Site of ISTH-defined major bleeding events among patients who experienced bleeding.
