# Supplementary material for: Sepsis-Induced Coagulopathy and Hypoalbuminemia: Endothelial Damage as Common Pathway and Clinical Implications on Mortality and Transfusion Risk
Source: J Clin Med. 2025 Jun 24;14(13):4483. doi: 10.3390/jcm14134483 (PMC12250473; doi:10.3390/jcm14134483)
Supplement: Supplementary file 1 [file jcm-14-04483-s001.zip › jcm-3701226-supplementary.pdf]

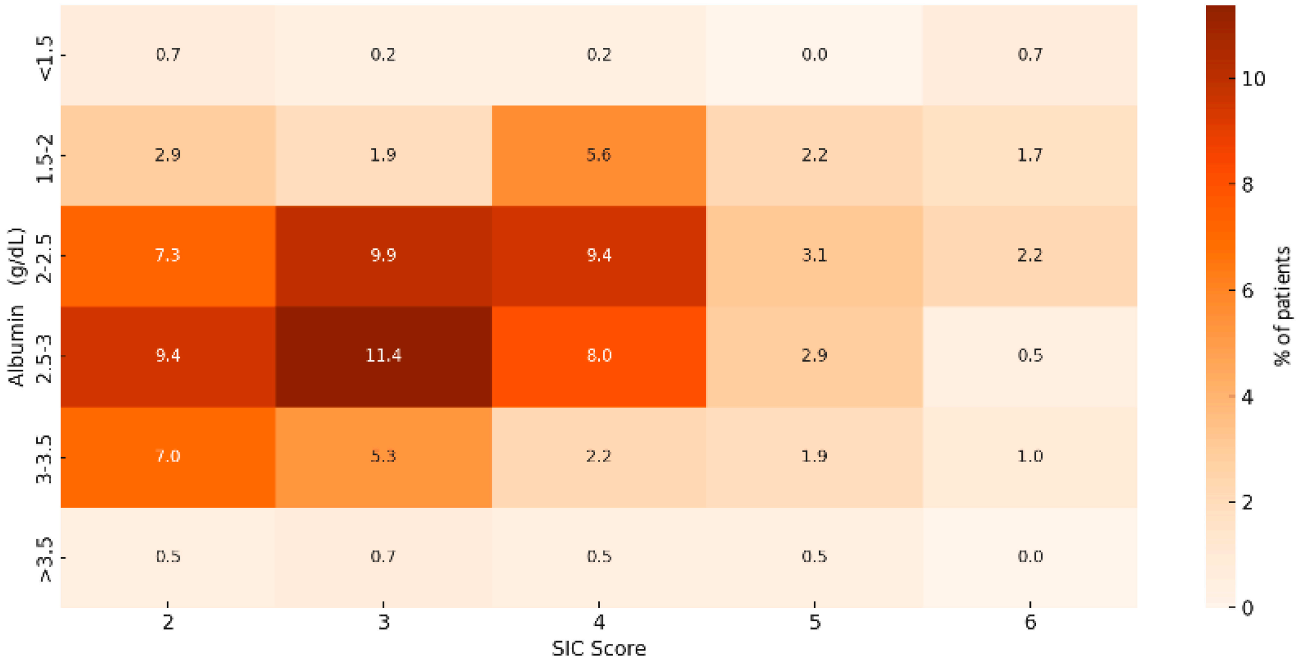

Supplementary Figure S1: A heatmap showing the percentage distribution of patients according to the SIC score and serum albumin levels categorized by clinical cut-offs. Each cell represents the proportion (%) of patients within a specific SIC score (x-axis) and albumin range (y-axis). The figure highlights the frequent overlap between moderate hypoalbuminemia and intermediate SIC scores, suggesting a clustering of patients in the mid-range of both variables. The color intensity is proportional to the relative frequency of patients in each cell.

Variable	Total	No SIC	SIC	p-value
Patients, n (%)	413 (100)	337 (81.6)	76 (18.4)	
Age, years, mean (SD)	71.5 (12.6)	71.4 (12.8)	72.3 (11.6)	0.579
Sex, n (%)				0.190
Male	153 (37)	130 (38.6)	23 (30.3)	
Female	260 (63)	207 (61.4)	53 (69.7)	
BMI, Kg/m2, median (IQR)	25.3 (22.8-28.4)	25.6 (23.1-28.6)	24.1 (21.6-27.4)	0.049
Comorbidities, n (%)				
Hypertension	264 (63.9)	227 (67.4)	37 (48.7)	0.003
Ischemic heart disease	54 (13.1)	47 (13.9)	7 (9.2)	0.347
Peripheral vascular disease	66 (16)	56 (16.6)	10 (13.2)	0.603
Stroke or TIA	34 (8.2)	29 (8.6)	5 (6.6)	0.652
Chronic heart failure	62 (15)	53 (15.7)	9 (11.8)	0.479
Diabetes	101 (24.9)	91 (27)	10 (14.7)	0.032
Chronic kidney failure	74 (17.9)	61 (18.1)	13 (17.1)	1.000
Active cancer	79 (19.1)	54 (16)	25 (32.9)	0.002
Charlson Comorbidity Index, point, mean (SD)	4.9 (2.6)	4.8 (2.6)	5.4 (2.5)	0.072
Antithrombotic therapy, n (%)				
Antiplatelet	75 (18.2)	67 (19.9)	8 (10.5)	0.069
Anticoagulant	120 (29.1)	91 (27)	29 (38.2)	0.068
Type of anticoagulant, n (%)				0.038
Direct oral anticoagulants	106 (88.3)	82 (90.1)	24 (82.8)	
Vitamin K antagonist	9 (7.5)	4 (4.4)	5 (17.2)	
Low molecular weight heparin	5 (4.2)	5 (5.5)	0 (0)	
Site of infection, n (%)				<0.001

Genitourinary	90 (21.8)	73 (21.7)	17 (22.2)	
Respiratory	173 (42)	154 (45.8)	19 (25)	
Intra-abdominal	46 (10.9)	39 (11.5)	6 (8.3)	
Musculoskeletal/skin	26 (6.3)	24 (7.1)	2 (2.8)	
Other	9 (2.3)	7 (2.2)	2 (2.8)	
Unknown/viral	69 (16.7)	40 (11.8)	30 (38.9)	
Vital signs				
RR, breaths/min, median (IQR)	20 (18-25)	20 (18-25)	20 (18-24)	0.458
SpO_2, %, median (IQR)	95 (92-98)	95 (92-97)	96 (94-98)	0.013
HR, bpm, median (IQR)	92 (80-108)	90 (80-108)	95 (81-108)	0.206
Systolic BP, mmHg, mean (SD)	113.4 (25.6)	115.9 (25.7)	102.6 (22.7)	<0.001
NEWS, point, mean (SD)	5.4 (3.5)	5.4 (3.5)	5.4 (3.4)	0.986
Inflammatory markers, median (IQR)	14.9 (7.9–22.3)	14.7 (7.7-22.1)	15.7 (9.6-23.5)	0.490
CRP, mg/dL	12.3 (7.4-17.7)	12.8 (8.2-18.3)	8.9 (2.9-16.2)	<0.001
WBC, $x10^9/L$	4.1 (0.9-20.5)	3.2 (0.8-16.1)	12.7 (1.2-41.4)	0.003
PCT, $x10^9/L$				
Coagulation parameters, median (IQR)	171 (115-251)	197 (142-271)	82 (50-107)	<0.001
Platelet count, $x10^9/L$	1.26 (1.15-1.41)	1.22 (1.13-1.33)	1.54 (1.33-1.89)	<0.001
PT-INR	1.23 (1.09-1.43)	1.21 (1.06-1.36)	1.45 (1.25-1.83)	<0.001
aPTT	538 (422-738)	557 (433-761)	450 (324-571)	<0.001
Fibrinogen, mg/dL	2151 (1076-4251)	2104 (1037-4214)	2871 (1349-7133)	0.128
D-Dimer, ng/mL				
Complete blood count				
Hb, g/dL, mean (SD)	11.9 (2.6)	12.2 (2.4)	10.7 (2.8)	<0.001
Hct, %, median (IQR)	0.37 (0.31-0.41)	0.37 (0.32)	0.32 (0.26-0.39)	<0.001

Creatinine, mg/dL, median (IQR)<br>Bilirubin, mg/dL, median (IQR)	1.46 (1.01-2.22)<br>0.9 (0.6-1.6)	1.51 (1.01-2.22)<br>0.9 (0.5-1.6)	1.41 (1.03-2-25)<br>1.2 (0.8-2.1)	0.981<br>0.001
Albumin, g/dL, mean (SD)	2.58 (0.52)	2.61 (0.51)	2.48 (0.56)	0.056
Severity scores, mean (SD)<br>SOFA score<br>APACHE II score	 4.2 (1.8)<br>12.6 (4.9)	 3.9 (1.7)<br>12.5 (4.9)	 5.5 (1.9)<br>13.3 (4.8)	 <0.001<br>0.196

Supplementary Table S1: Baseline characteristics of the study population stratified by SIC status. *SIC: sepsis-induced coagulopathy. BMI: body mass index. CHF: congestive heart failure. CKD: chronic kidney disease. RR: respiratory rate. SpO_2: peripheral oxygen saturation. HR: heart rate. SBP: systolic blood pressure. NEWS: National Early Warning Score. CRP: C-reactive protein. WBC: white blood cell count. PLT: platelet count. PT-INR: prothrombin time–international normalized ratio. aPTT: activated partial thromboplastin time. Hb: hemoglobin. Hct: hematocrit. SOFA: Sequential Organ Failure Assessment. APACHE II: Acute Physiology and Chronic Health Evaluation II.*

Variables	No ISTH-defined major bleeding event	ISTH-defined major bleeding event	p-value
Patients, n (%)	352 (85.2)	61 (14.8)	
Age, years, mean (SD)	71.9 (12.9)	69.4 (10.7)	0.142
Sex, n (%)			0.250
Male	126 (35.8)	27 (44.3)	
Female	226 (64.2)	34 (55.7)	
BMI, Kg/m2, median (IQR)	25.7 (23.4-28.7)	23.3 (21.8-26.9)	0.003
Comorbidities, n (%)			
Hypertension	232 (65.9)	32 (52.5)	0.060
Ischemic heart disease	46 (13.1)	8 (13.1)	1.000
Peripheral vascular disease	58 (16.5)	8 (13.1)	0.576
Stroke or TIA	27 (7.7)	7 (11.5)	0.315
Chronic heart failure	54 (15.1)	8 (13.1)	0.846
Diabetes	92 (26.6)	9 (15.3)	0.073
Chronic kidney failure	60 (17)	14 (23)	0.279
Active cancer	54 (15.3)	25 (41)	<0.001
Charlson Comorbidity Index, point, media (SD)	4.9 (2.6)	5.3 (2.2)	0.229
Antithrombotic therapy, n (%)			
Antiplatelet	66 (18.8)	9 (14.8)	0.589
Anticoagulant	101 (28.7)	19 (31.1)	0.760
Type of anticoagulant, n (%)			0.001
Direct oral anticoagulants	94 (93.1)	12 (63.2)	
Vitamin K antagonist	5 (5.0)	4 (21.1)	
Low molecular weight heparin	2 (2)	3 (15.8)	
Site of infection, n (%)			0.002

Genitourinary	81 (22.9)	9 (15.3)	
Respiratory	153 (43.5)	21 (33.9)	
Intra-abdominal	40 (11.3)	5 (8.5)	
Musculoskeletal/skin	18 (5.1)	8 (13.6)	
Other	8 (2.4)	1 (1.7)	
Unknown/viral	52 (14.9)	17 (27.1)	
Vital signs			
RR, breaths/min, median (IQR)	20 (18-25)	20 (18-22)	0.032
SpO_2, %, median (IQR)	95 (92-97)	96 (93-98)	0.067
HR, bpm, median (IQR)	93 (80-108)	88 (79-105)	0.546
Systolic BP, mmHg, mean (SD)	115.1 (25.8)	103.6 (22.7)	0.001
NEWS, point, mean (SD)	5.5 (3.5)	4.9 (3.1)	0.213
Inflammatory markers, median (IQR)			
CRP, mg/dL	14.8 (7.7-22.6)	16.1 (10.5-19.5)	0.532
WBC, $x10^9/L$	12.5 (7.9-17.9)	10.4 (4.6-15.5)	0.053
PCT, $x10^9/L$	3.7 (0.8-18)	7.1 (1.63-32.7)	0.040
Coagulation parameters, median			
(IQR)	173 (124-258)	131 (67-229)	0.006
Platelet count, $x10^9/L$	1.25 (1.14-1.39)	1.32 (1.23-1.65)	0.005
PT-INR	1.22 (1.08-1.41)	1.36 (1.17-1.56)	0.008
aPTT	541 (422-761)	520 (412-615)	0.193
Fibrinogen, mg/dL	2065 (1108-4110)	2897 (1108-5901)	0.081
D-Dimer, ng/mL			
Complete blood count			
Hb, g/dL, mean (SD)	12.4 (2.3)	9.3 (2.3)	<0.001
Hct, %, median (IQR)	0.37 (0.33-0.42)	0.28 (0.24-0.32)	<0.001
Creatinine, mg/dL, median (IQR)	1.44 (1.01-2.15)	1.73 (1.15-3.22)	0.036

Bilirubin, mg/dL, median (IQR)	1 (0.6-1.7)	0.9 (0.5-1.5)	0.378
Albumin, g/dL, mean (SD)	2.63 (0.52)	2.32 (0.45)	<0.001
SIC score, point, mean (SD)	3.3 (1.1)	3.9 (1.3)	<0.001
Severity scores, mean (SD)			
SOFA score	4.1 (1.8)	4.7 (1.9)	0.018
APACHE II score	12.3 (4.8)	14.4 (4.6)	0.016

Supplementary Table S2: Baseline characteristics of the study population stratified by the presence of ISTH-defined major bleeding events. *SIC: sepsis-induced coagulopathy. BMI: body mass index. CHF: congestive heart failure. CKD: chronic kidney disease. RR: respiratory rate. SpO_2: peripheral oxygen saturation. HR: heart rate. SBP: systolic blood pressure. NEWS: National Early Warning Score. CRP: C-reactive protein. WBC: white blood cell count. PLT: platelet count. PT-INR: prothrombin time–international normalized ratio. aPTT: activated partial thromboplastin time. Hb: hemoglobin. Hct: hematocrit. SOFA: Sequential Organ Failure Assessment. APACHE II: Acute Physiology and Chronic Health Evaluation II.*

**Site**	**n (%)**
Hematuria	4 (6.5)
Consumption/microangiopathic anemia	27 (44.3)
Gastrointestinal Bleeding	11 (18.1)
Cutaneous/muscular bleeding	5 (8.1)
Hemothorax/hemoperitoneum	3 (4.9)
Hematologic disease-related bleeding	11 (18.1)

Supplementary Table S3: Site of ISTH-defined major bleeding events among patients who experienced bleeding
